# Patient Satisfaction With General Anesthesia Compared to Spinal Anesthesia for Cesarean Section: A Multicenter Observational Study

**DOI:** 10.7759/cureus.42666

**Published:** 2023-07-29

**Authors:** Suzana Sobot Novakovic, Sanja Cuk, Dragan Svraka, Dragan Milosevic

**Affiliations:** 1 Anesthesiology and Critical Care, University Clinical Center of Republic of Srpska, Banja Luka, BIH; 2 Anesthesiology and Critical Care, University Clinical Center of the Republic of Srpska, Banja Luka, BIH

**Keywords:** parturients, general anesthesia, anesthesia spinal, obstetric anesthesia, patient satisfaction

## Abstract

Background

Satisfaction in patients undergoing cesarean section (CS) is of great importance in every hospital. There are big differences between spinal and general anesthesia for CS in terms of outcome, recovery times, and quality of service.

Methods

This multicenter observational study included 1443 patients who had elective (n=622) or emergency (n=821) CS in five medical centers over the period of 16 months. Bauer questionnaire was used for measuring patient satisfaction after CS. The questionnaire contained 15 questions about anesthesia-related discomfort and satisfaction with anesthesia care.

Results

During the study period, 1161 (80%) patients underwent CS under general anesthesia (GA) and 282 of them (20%) received spinal anesthesia (SA) for CS. The most frequently reported anesthesia-related discomfort was pain at the surgical site (>70%), drowsiness (68%), and thirst (60%). The data on patient satisfaction showed high satisfaction that exceeded 90%. Anesthesia side effects were less frequent and the general satisfaction rate was higher in the SA group compared to the GA group (*P* < .001).

Conclusion

SA for CS had less frequent side effects and a better satisfaction rate compared to GA for CS. Hospitals need to make room for improvement of postoperative acute pain control and introduction to Enhanced Recovery After Surgery (ERAS) protocols for elective CS.

## Introduction

Patient satisfaction refers to a patient's overall perception of the care they received from a healthcare provider or facility. It is a measure of how well the healthcare system meets the patient's expectations and needs. Patient satisfaction can be influenced by many factors and healthcare providers and facilities often use patient satisfaction surveys to gather feedback from patients and identify areas for improvement [1-4]. Patient satisfaction in pregnant patients is a big concern for all health professionals. These patients may experience sensitivity due to hormonal changes, physical discomfort, and emotional stress. It is important to provide them with compassionate care, respect their privacy, and listen to their concerns. High levels of pregnant patient satisfaction are associated with better health outcomes for both the mother and the baby [5]. This may include providing them with extra support, offering pain management options, and being attentive to their needs during labor and delivery. It is important to treat pregnant patients with care and sensitivity to ensure a positive healthcare experience for both the patient and the healthcare provider [6].

Cesarean section (CS) is a surgical procedure where a neonate is delivered through an incision on the abdominal wall and uterus of the mother. It is used in situations where vaginal delivery is not possible and often refers to as a lifesaving procedure. Regional and general anesthesia are commonly used for CS and both have their advantages and disadvantages. Regional anesthesia is generally preferred as a type of anesthesia for CS but also general anesthesia is still frequently used in some countries, largely due to greater familiarity with it [7]. The type of anesthesia and anesthesia management for CS largely affects pregnant patient satisfaction. Many studies compared differences between these two types of anesthesia for CS but mainly for neonatal and maternal outcomes [8,9]. Not many of them compared patient satisfaction [10]. 

The primary objective of the study was to determine patient satisfaction with anesthesia care and the incidence of anesthesia-related discomfort after CS. A secondary objective was to compare the satisfaction and anesthesia-related discomfort in patients who underwent general anesthesia compared to those who received spinal anesthesia for CS.

## Materials and methods

The study was submitted for approval by the Ethics Committee of the University Clinical Centre of the Republic of Srpska (UCC RS) on October 7, 2016, and received certificate No: 01-9-670.2/16. This was a multicenter, prospective, observational study performed in the period from November 2016 to February 2018. The study included 1443 patients who underwent CS and gave informed consent to participate in the research. The investigation was carried out in the Republic of Srpska (RS), Bosnia-Herzegovina (BIH), in five medical centers that agreed to be involved. The same study group was used for examining the incidence of accidental awareness under general anesthesia (AAGA) using the Brice questionnaire and the results of this research were published on April 4, 2023 [11]​​​​​**.**

All adults over 18 years of age with a CS delivery and an ASA (American Society of Anesthesiologists) physical status of 2 or 3 were included in the study. Criteria for exclusion were: refusal to participate, incomplete questionnaires, subjects who later asked to be excluded from the trial, newborn death, and all subjects with severe perioperative hemorrhage. In addition, patients whose psychological state or state of consciousness prevented them from speaking and completing the questionnaire were excluded. The study protocol did not standardize general or regional anesthesia for CS. Induction to general anesthesia was performed with thiopental (3-5 mg/kg) or propofol (1,5-2,5 mg/kg) and sustained with sevoflurane (MAC1). Intubation relaxation was performed with succinylcholine (1,5 mg/kg) and sustained with atracurium or pancuronium as needed. Fentanyl was used to maintain intraoperative analgesia. Isobaric 0.5% bupivacaine (10-13 mg) was utilized for spinal anesthesia.

In each hospital, a researcher was in charge of coordinating data gathering. Patient demographic information was collected for every patient. Bauer questionnaire [4] was employed for measuring patient satisfaction after CS. The questionnaire consisted of 15 questions (Appendices). The first set of questions was attributed to anesthesia-related discomfort and the second one to satisfaction with anesthesia care. The answers to the questions on anesthesia-related discomfort were graded as: no/yes, moderate/yes, or severe. The questions on satisfaction with anesthesia care were given on a four-item scale (very satisfied/satisfied/dissatisfied/very dissatisfied). According to the conditions in some hospitals, subjects were interviewed by the investigator, who completed the questionnaires while speaking to the subjects, or the subjects received the questionnaire and completed it themselves without speaking to the investigator. Subjects were interviewed or completed questionnaires 24 hours after surgery.

Statistical analysis

Data analysis was done using SPSS software (IBM Corp. released in 2011. IBM SPSS Statistics for Windows, Version 21.0. Armonk, NY: IBM Corp). Graphs and tables are utilized to display the findings. Relative and absolute frequencies are used for presenting categorical data. Standard deviation (SD) and arithmetic mean are employed for describing numerical data. The non-parametric χ² test was employed to determine the relationship between the patients' satisfaction and the type of anesthesia they underwent, and when the necessary conditions for its application were not met, cross-tabulations were used.

## Results

The 16-month study involved 1,518 subjects, of whom 1,443 (95.1%) were eligible for participation. Subsequently, a total of 75 patients (4.9%) were excluded from the investigation. General information about patients and surgery is presented in Table 1. During the study period, 1161 (80%) patients underwent CS under general anesthesia (GA) and 282 of them (20%) received spinal anesthesia for CS. The two groups were similar in terms of demographic data, chronic illnesses, and therapy. Statistically significantly more patients underwent general anesthesia compared to spinal. Statistically significantly more emergency CS have been performed under general compared to spinal anesthesia (Table 1).

**Table 1:** Data on patient characterization, anesthesia, surgery by urgency NA – not applicable; SD – standard deviation; G – general anesthesia; S – spinal anesthesia; P – probability; ASA - American Society of Anesthesiologists physical status classification system

Variables	Group G (n=1161)	Group S n=282	All patients n=1443	P
Age (mean + SD)	31.7 (SD 5.32)	31.8 (SD 5.14)	31.28 (SD 5.29)	NA
Age groups (%)				
< 25 years old	14.2	12.5	13.8	0.213
26-30 years old	31	26.7	30.2	
31-35 years old	32.7	35.2	33.2	
>35 years old	22.1	25.6	22.8	
Education n (%)				
Without formal education	6 (0.5)	0	6 (0.4)	0.220
Elementary school	37 (3.3)	5 (1.8)	42 (3)	
High school education	657 (58.3)	148 (52.7)	805 (57.2)	
Higher education	417 (37)	125 (44.5)	542 (38.5)	
Postgraduate education	10 (0.9)	3 (1.1)	13 (0.9)	
ASA score n (%)				
2	1151 (99.1)	281 (99.6)	1432 (99.2)	0.157
3	10 (0.9)	1 (0.4)	11 (0.8)	
Surgery type based on urgency n (%)				
Elective CS	427 (36.7)	195 (69.1)	622 (43.2)	<0.001
Emergency CS	734 (63.3)	87 (30.9)	821 (56.8)	
Interviewees with chronic diseases n (%)	291 (25.1)	71 (25)	362 (25.1)	NA
Interviewees with chronic therapy n (%)	223 (19.2)	57 (20.1)	280 (19.2%)	NA

Anesthesia-related discomfort

**Figure 1:**
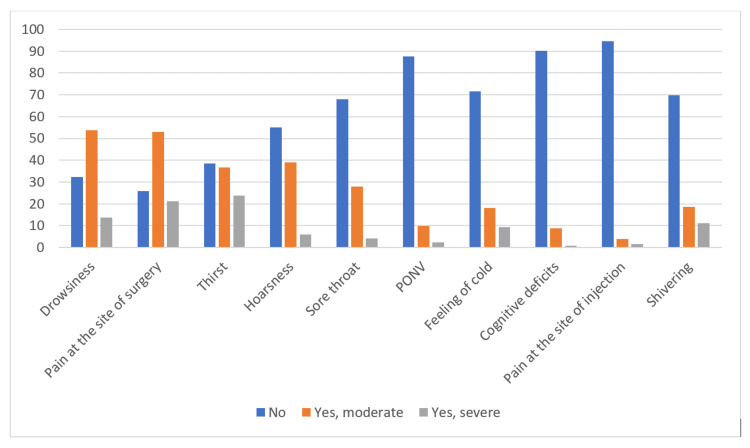
Distribution of answers to the questions about anesthesia-related discomfort Anesthesia-related discomfort reported by the patients; PONV - postoperative nausea and vomiting

The distribution of answers to anesthesia-related discomfort is presented in Figure 1. As the major source of discomfort, patients stated pain at the site of the surgical incision, drowsiness, and thirst. Over 70% of patients reported having pain after surgery and a fifth of these patients reported that the pain was severe. Drowsiness was reported by 68% of patients, among whom more than 50% stated that the drowsiness was moderate. Thirst was reported by 60% of patients and almost a quarter stated that the thirst was severe. Hoarseness was reported by over 40% of patients, of which only 5% reported that it was severe. Sore throat, nausea and vomiting, feeling cold, concentration problems, pain at the site of anesthetic administration, and shivering were reported by less than 30% of the patients.

Statistically significant differences were found in the analysis of anesthesia-related discomfort and their comparison between the groups. Drowsiness, pain at the site of the surgical incision, thirst, hoarseness, and sore throat were statistically significantly higher in the GA group compared to the SA group. The feeling of cold, pain at the site of injection, and shivering are statistically significantly higher in the SA group (Table 2). The incidence of postoperative nausea and vomiting (PONV) was less than 20% but with a slightly higher number of patients in the SA group. The calculated value of the Cramer's V = .094 tells us that there is a weak connection between the variables. Cognitive deficit was reported by a smaller number of patients in both groups. Slightly higher number of patients with moderate symptoms of cognitive deficit were in GA group compared to SA group. The calculated value of the Cramer's V = .083 tells us that there is a weak connection between the variables (Table 3).

**Table 2:** The most common anesthesia-related discomfort reported by the patients* – comparison between the groups n – number of patients; χ² - chi-square value; *P* - probability; G – general anesthesia; S – spinal anesthesia. *anesthesia-related discomfort reported in ≥ 30% of the patients

Anesthesia-related discomfort	Group G (n=1161) n (%)	Group S (n=282) n (%)	Total (n=1443) n (%)	χ²	P
Drowsiness					
no	284 (24,5)	186 (65.9)	470 (32,5)	190.168	<0.001
yes, moderate	691 (59.5)	85 (29.7)	775 (53,7)		
yes, severe	186 (16)	11 (3.9)	197 (13,6)		
Pain at the site of surgery					
no	269 (23,1)	108 (37.9)	376 (26.1)	27.995	<0.001
yes, moderate	632 (54.4)	130 (46.1)	762 (52.8)		
yes, severe	260 (22.4)	44 (15.6)	304 (21.1)		
Thirst					
no	410 (35.3)	150 (53.2)	560 (38.8)	34.605	<0.001
yes, moderate	448 (38.6)	86 (30.1)	533 (36.9)		
yes, severe	303 (26.1)	46 (16.3)	349 (24.1)		
Hoarseness					
no	565 (48.7)	232 (82.3)	797 (55,2)	112.045	<0.001
yes, moderate	519 (44.7)	42 (14.8)	561 (38.8)		
yes, severe	77 (6.6)	8 (3.2)	85 (5.9)		
Sore throat					
no	729 (62,8)	255 (90.4)	984 (68.2)	90.277	<0.001
yes, moderate	379 (32.7)	20 (7.1)	399 (27.6)		
yes, severe	52 (4.5)	7 (2.5)	59 (4.1)		

**Table 3:** The less common anesthesia side effects reported by the patients* – comparison between the groups n – number of subjects; χ² - chi-square test; *P* - probability; G – general anesthesia; S – spinal anesthesia; PONV – postoperative nausea and vomiting. *anesthesia-related discomfort reported in ≤ 30% of subjects

Anesthesia related discomfort	Group G (n=1161) n (%)	Group S (n=282) n (%)	Total (n=1443) n (%)	χ²	P
PONV					
no	1042 (89.7)	228 (80.8)	1270 (88.0)	12.720	0.002
yes, moderate	104 (8.9)	40 (14.1)	144 (9.9)		
yes, severe	15 (1.3)	14 (4.9)	29 (2)		
Feeling of cold					
no	874 (75.4)	162 (57.4)	1036 (71.8)	31.892	<0.001
yes, moderate	192 (16.5)	75 (26.5)	267 (18.5)		
yes, severe	93 (8)	45 (15.9)	138 (9.5)		
Cognitive deficit					
no	1043 (89.9)	260 (95.9)	130 (91.1)	9.946	0.007
yes, moderate	114 (9.8)	11 (4.1)	0		
yes, severe	3 (0.2)	11 (3.3)	4 (0.2)		
Pain at the site of injection					
no	1120 (96.4)	249 (88.3)	1369 (94.8)	18.393	<0.001
yes, moderate	33 (2.8)	26 (9,2)	59 (4.1)		
yes, severe	8 (0.7)	7 (2.4)	15 (1)		
Shivering					
no	862 (74.2)	148 (52.4)	1010 (69.9)	46.850	<0.001
yes, moderate	188 (16.1)	84 (29.7)	272 (18.8)		
yes, severe	111 (9.5)	50 (17.7)	161 (11.1)		

Satisfaction with anesthesia care

**Figure 2:**
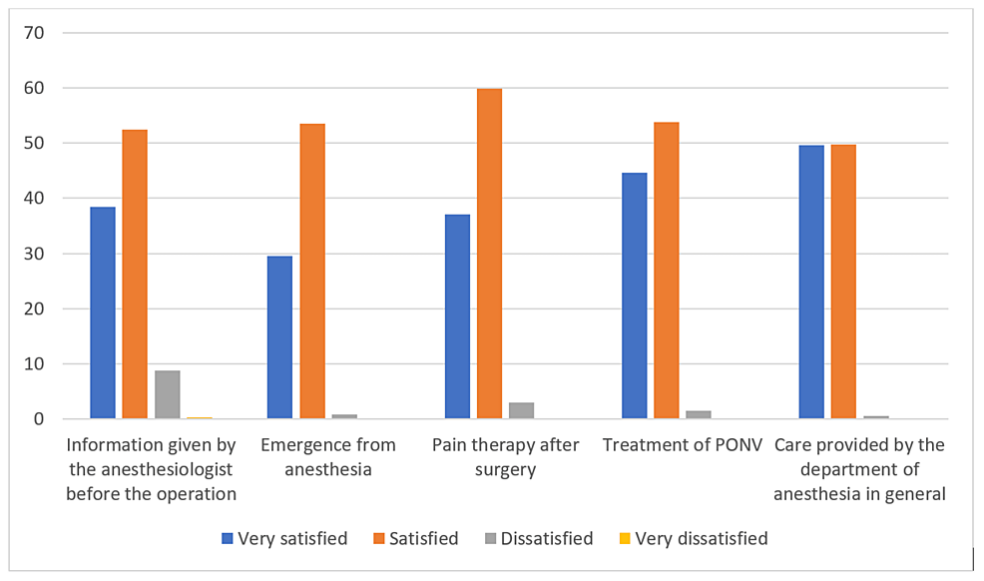
Distribution of answers to the questions related to satisfaction with anesthesia care Satisfaction with anesthesia care reported by the patients PONV: postoperative nausea and vomiting

In the second part of the interview, patients expressed their satisfaction with anesthesia care. Most of the interviewees were satisfied with the treatment they were given by the anesthesiology team. Over 90% of subjects claimed to be satisfied with the information they were given about anesthesia preoperatively, control of pain, PONV, and overall treatment by the anesthesiology team (Figure 2). Analysis of the relationship between satisfaction with anesthesia care and the type of anesthesia was not possible due to the small number of interviewees who stated they were "dissatisfied" and "very dissatisfied". Therefore, comparisons were made between the SA and GA groups among the patients who stated they were "satisfied" and "very satisfied" (Table 4). Statistically significant differences between the groups were found. Patients in the SA group expressed higher satisfaction with the anesthesia care compared to the patients in the GA group.

**Table 4:** The relationship between satisfaction with anesthesia care and the type of anesthesia n – number of subjects; χ² - chi-square value; P – probability; G – general anesthesia; S – spinal anesthesia. *Most of the subjects in the SA group did not answer the question about emergence from anesthesia.

Satisfaction with anesthesia care	Group G (n=1161) n (%)	Group S (n=282) n (%)	Total (n=1443) n (%)	χ²	P
How satisfied were you with the information you were given by the anesthesiologist before the operation?					
Very satisfied	376 (32.4)	178 (63.1)	554 (38.4)	76.846	<0.001
Satisfied	665 (57.3)	92 (32.6)	757 (52.5)		
Dissatisfied	117 (10)	10 (3.5)	127 (8.8)		
Very dissatisfied	3 (0.3)	2 (0.7)	5 (0.34)		
How satisfied were you with emergence from anesthesia?					
Very satisfied	386 (33.2)	39 (13.8)*	425 (29.4)	37.328	<0.001
Satisfied	762 (65.6)	12 (4.2)*	774 (53.6)		
Dissatisfied	12 (1)	0*	12 (0.8)		
Very dissatisfied	1 (0.1)	0*	2 (0.2)		
How satisfied have you been with pain therapy after surgery?					
Very satisfied	384 (33.1)	152 (53.9)	536 (37.1)	47.561	<0.001
Satisfied	749 (64.5)	115 (40.7)	864 (59.9)		
Dissatisfied	27 (2.3)	15 (5.3)	42 (2.9)		
Very dissatisfied	1 (0.1)	0	1 (0.06)		
How satisfied were you with treatment of nausea and vomiting after the operation?					
Very satisfied	486 (41.9)	157 (55.7)	643 (44.5)	18.609	<0.001
Satisfied	659 (56.8)	118 (41.8)	777 (53.8)		
Dissatisfied	15 (1.3)	6 (2.1)	21 (1.5)		
Very dissatisfied	1 (0.08)	1 (0.4)	2 (0.1)		
How satisfied were you with the care provided by the department of anesthesia in general?					
Very satisfied	515 (44.3)	200 (70.9)	715 (49.5)	64.915	<0.001
Satisfied	640 (55.1)	79 (28)	719 (49.8)		
Dissatisfied	4 (0.3)	3 (1.1)	7 (0.5)		
Very dissatisfied	2 (0.2)	0	2 (0.1)		

## Discussion

With this study, we aimed to evaluate patient satisfaction after CS and the incidence of anesthesia-related discomfort with their comparisons between groups where SA and GA were applied for CS.

Anesthesia-related discomfort

Most of the interviewees complained about pain at the site of surgery, drowsiness, and thirst. All of these three adverse effects were statistically significantly higher in the group of patients who received GA. Pain is the discomfort that the majority of interviewees complained about after the surgery. 73% of patients reported pain after the surgery and 20% of them even had severe pain. This result is similar to those with Bauer et al. [4]. When looking at the groups, significantly fewer patients complained of pain in the SA group (60%) compared to the GA group (76%). But irrespective of the applied anesthesia pain is still a major issue after CS. Our results are similar to previous studies [12,13] which reported a high incidence of postoperative pain after CS with incidence between 65% and 70%. These data indicate the necessity of establishing an acute pain service in our institutions in order to reduce pain and improve quality.

Drowsiness is the second most common complaint of patients after CS. It was reported by 66% of the patients in the current study. 76% of patients in the GA group reported this discomfort and 16% of them even complained that the drowsiness was severe. Although drowsiness is not expected to be significantly reported by the subjects under SA, 30% of those patients in the current study asserted that they felt this way after the surgery. Earlier studies also reported a high incidence of drowsiness after GA, which is one of the three most reported side effects after anesthesia. Bauer et al. reported an incidence of drowsiness of 80%, which was one of the most frequent discomforts reported by subjects in this investigation [4]. Walker et al. reported drowsiness in 64% of patients, and 10% of those claimed that this side effect of anesthesia was severe [14]. This is a subjective feeling and some patients find it difficult to make gradation and evaluate its origin. Drowsiness with its symptoms may be similar to postoperative fatigue. Postoperative fatigue depends on the degree of surgical trauma. It is mediated by the endocrine-metabolic response to surgery, impaired nutritional intake in the postoperative period, and immobilization [15]. This could explain why 30% of our patients reported this discomfort after spinal anesthesia even though they were not exposed to general anesthetics.

The use of opioids in the postoperative period for analgesia may also affect the occurrence of drowsiness after surgery [16]. Thirst is one of the frequent complaints reported by patients after surgery [4,14,17]. In the current study, this discomfort was reported with an incidence of 60%. Patients in the SA group reported less drowsiness (45%) compared to the GA group (64%). Prolonged fasting in patients before elective CS due to the risk of regurgitation and aspiration often leads to relative dehydration before surgery. Parturients who are in the maternity ward are often deprived of oral intake of liquids and food before surgery. This also contributes to the onset of dehydration and symptoms of thirst in the postoperative period for those who ended up in emergency CS [18,19]. Introducing the Enhanced Recovery After Surgery (ERAS) protocol and liberalizing fluid intake during childbirth would significantly reduce symptoms of dehydration and increase patient satisfaction [20].

Satisfaction with anesthesia care

Results of the current study indicate a high degree of patient satisfaction. Over 90% of patients were satisfied with anesthesia for CS. This observation where a major proportion of the patients expressed their high level of satisfaction was pointed out by previous studies [21]. For this reason, Bauer et al. emphasized the importance of making a type of questionnaire where questions were given on a type of grading scale. By grading answers as "satisfied" and "very satisfied" we can easily identify a lower level of satisfaction among patients and discover areas that require improvement. With the Bauer questionnaire, we were able to discover significant differences in service quality and patient satisfaction after CS. Only about 30% of patients who were in GA were "very satisfied" with information about anesthesia, awakening from anesthesia, and pain control, and about 40% of them were "very satisfied" with PONV control and the care provided by the anesthesiology team. These results indicate that there is considerable room for quality improvement. On the other hand, patients in the SA group had significantly greater satisfaction, with around 60% of them being "very satisfied" with the information they received about anesthesia preoperatively, over 50% of them were "very satisfied" with pain control and PONV, and 70% of them were "very satisfied" with the care provided by the anesthesia team. These statistics indicate a much higher degree of satisfaction among patients who underwent SA for CS.

The study has certain limitations and potential sources of error. It is an observational study, so many conditions are not controlled by the study protocol. Some patients were interviewed in a direct conversation with the researcher and some filled out the questionnaires independently. There is a much higher number of subjects in the group with GA compared to the group of patients with SA. There is a non-uniform distribution of patients regarding the type of surgery based on urgency. There is a much higher number of subjects in the SA group who underwent elective CS compared to the GA group, where more patients underwent emergency CS. This uneven distribution certainly affected the results of the study and requires observation in future studies.

## Conclusions

There is a significant difference in patient satisfaction and side effect incidence between GA and SA for CS. Patients with SA for CS reported much higher satisfaction and a lower incidence of secondary effects compared to patients who received GA for CS. Questionnaires like the Bauer questionnaire have proven to be a good tool for assessing the quality of service and patient satisfaction. In our hospitals, there is room for quality improvement, especially regarding acute pain service and the introduction of the ERAS protocol for elective CS.

## Figures and Tables

**Figure 3:**
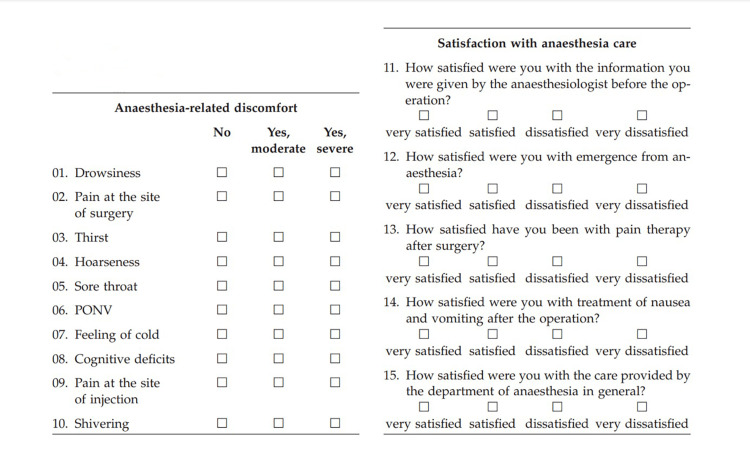
Bauer questionnaire - final set of questions The final set of questions used in Bauer et al. [4].
